# Learning to be unfaithful – preferences and flower constancy of *Eristalis tenax* (Syrphidae) decrease with experience

**DOI:** 10.1242/jeb.251711

**Published:** 2026-05-11

**Authors:** Jakub Štenc, Alice Haveldová, Eva Matoušková, Zdeněk Janovský

**Affiliations:** ^1^ Department of Botany, Faculty of Science, Charles University, Benátská 2, CZ-128 41 Prague, Czech Republic; ^2^ Department of Zoology, Faculty of Science, Charles University, Viničná 7, CZ-128 41 Prague, Czech Republic; ^3^ CREAF, Centre for Ecological Research and Forestry Applications, 08193, Bellaterra, Spain; ^4^ Svatý Jan t. Krsovice 1, CZ-285 04 Uhlířské Janovice, Czech Republic

**Keywords:** Artificial flowers, Plant–pollinator interaction, Plant specialization, Reward, Hoverflies, Pollination

## Abstract

Pollinators exhibit preferences and flower constancy (i.e. the proportion of visits to the same plant species) toward particular floral traits, which drive their decisions while foraging between flowers. Furthermore, pollinators adjust their preferences and flower constancy based on their experience with the flower reward, shaping their foraging behaviour and consequently pollen transfer. However, while evidence of the pollinators learning from foraging is well known for bees, evidence for hoverflies is only slowly increasing although they represent one of the most abundant pollinator groups in temperate zones. We compared the preferences and flower constancy of flower naive and experienced individuals of the drone fly *Eristalis tenax* in controlled, full-factorial experiments using artificial flowers differing in colour and size. Our results show that previous experience with flowers in an environment rich in flowering species decreased flower constancy and changed the preferences of *E. tenax*. We discuss the effect of changes in preference and constancy on *E. tenax* foraging among flowering plants.

## Teaser

**Summary:** Foraging experience reduces flower constancy: wild drone flies display lower constancy and altered preferences compared with naive individuals, revealing a shift toward opportunistic strategies in species-rich plant communities.

## INTRODUCTION

Pollinators foraging between flowers of the same species are crucial for pollen transfer and, consequently, sexual reproduction of the majority of vascular plant species (Ollerton et al., 2011), with direct implications for plant evolution and diversification (van der Niet and Johnson, 2012; van der Niet et al., 2014). Yet, from a pollinator perspective, it is beneficial to optimise for nutrient and energy intake obtained during foraging (Pyke, 1978, 2019), which often involves visiting multiple plant species during a foraging bout (Kunin and Iwasa, 1996) and, consequently, to heterospecific pollen transfer (Briggs et al., 2016). However, it may be beneficial for pollinators to choose flowers based on their traits, signalling the presence of a reward (Wright and Schiestl, 2009; Krishna and Keasar, 2018). Plants may then benefit from developing traits that attract pollinators, increasing the probability of visits (Jersáková et al., 2012; van der Kooi et al., 2019) and deposition of conspecific pollen (Montgomery, 2009; Van der Niet et al., 2020). This may be even more beneficial in the species-rich community with predominantly generalist plant–pollinator interactions (Waser et al., 1996), where pollinator temporal specialisation is hypothesised to be crucial for successful pollen delivery to stigmas (Brosi, 2016; Bruninga-Socolar et al., 2023).

Pollinator behaviour during foraging among flowers is commonly described by two key terms: pollinator preference and flower constancy (Waser, 1986). Preference refers to how often a pollinator chooses one plant species or flower type over others, which can be influenced by flower traits or change depending on the availability and reward offered (Waser, 1986). Flower constancy occurs when a pollinator visits flowers of the same species (or type) in consecutive visits during the foraging bout, even when other types are available (Wells and Wells, 1986; Waser, 1986; Hill et al., 1997). While the benefit of flower constancy for pollen transfer is straightforward (Brosi, 2016), from a pollinator perspective, it may be sub-optimal as they may ignore flowers with equal or greater reward (Waser, 1986; Wells and Wells, 1986; Latty and Trueblood, 2020). Several hypotheses suggest that flower constancy helps pollinators by reducing the time needed to collect rewards, especially in complex environments, because of their sensory and memory limitations (Goulson, 2000; Raine and Chittka, 2007; Austin et al., 2019).

It has been demonstrated that some pollinators can change their preferences and modify flower constancy based on previous experience. For instance, individual bees tend to be less constant when differences in floral rewards are low (Chittka et al., 1999; Grüter et al., 2011; but see Hill et al., 1997) and preferences of hoverfly *Eristalis tenax* have recently been completely modified using appetitive conditioning with visual and chemical stimuli (Rajan et al., 2025). Pollinators are thought to be born with a set of innate preferences, navigating them toward flowers from which the reward is expected (Lunau and Maier, 1995; Gumbert, 2000); however, based on the experience with previously obtained reward, pollinators change their preference to some extent (Kelber, 2003; Weiss and Papaj, 2003) or even completely (Rajan et al., 2025). The learned preferences then can persist for days or weeks (Goulson and Cory, 1993; Rajan et al., 2025) and further affect pollinator foraging behaviour (Gumbert, 2000). Experimental evidence also shows that the increasing complexity (i.e. the number of possible choices) slows down bees' decisions and response to reward level changes (Austin et al., 2019). In contrast, studies on *E. tenax* show that complex multimodal signals (combining visual and chemical traits) facilitate the complete modification of innate preferences (Rajan et al., 2025). This may be important in a flower-rich environment with variable reward levels, where the cost of deciding among many choices may be higher, and pollinators may especially benefit from increased constancy toward already proven reward sources. But, if the reward intake and handling time do not differ significantly among the flowers, it is beneficial to pollinators to reduce flower constancy (Gegear and Thomson, 2004). However, our knowledge about the effect of learning on flower constancy is limited to a few pollinator taxa, with the majority of the research focused primarily on bees (e.g. Chittka et al., 1999; Austin et al., 2019), and to a lesser extent on butterflies (e.g. Goulson and Cory, 1993) and birds (e.g. Schmid et al., 2016).

Hoverflies are among the most common pollinators in Central Europe (Janovský and Štenc, 2023), often with a generalised foraging strategy (Klečka et al., 2018; Lucas et al., 2018; Doyle et al., 2020; Horiuchi et al., 2022), and exhibit varying levels of flower constancy (Goulson and Wright, 1998; Janovský et al., 2017). *Eristalis tenax* is particularly suited for manipulative studies because of its abundance and ease of rearing (Nicholas et al., 2018). Experiments have shown an innate preference, measured as landing or proboscis extension reflex (PER), in *E. tenax* for bright yellow (Lunau and Wacht, 1997; Dinkel and Lunau, 2001; An et al., 2018; Matoušková et al., 2023) and larger flowers (Matoušková et al., 2023), but fewer studies have explored learning and flower constancy in this species (e.g. Bennett, 1883; Lunau and Wacht, 1997; Ustinova and Lysenkov, 2020; Rajan et al., 2025). Lunau et al. (2018) have shown limited ability of learning in the context of PER; however, a recent study showed the ability of *E. tenax* to abandon their innate preferences of PER in favour of higher rewarding flowers in conditions using both visual (colour) and chemical (odour) stimuli (Rajan et al., 2025). Following previous findings, we investigated whether learning ability affects the foraging behaviour of *E. tenax* from natural plant–pollinator communities.

We hypothesised that experience with flowers differing in visual traits and reward availability shapes the foraging behaviour of *E. tenax*, particularly their preferences and flower constancy. To test this, we compared the foraging choices of flower naive (laboratory-reared) and experienced (wild-caught) individuals using artificial flowers in a fully factorial experiment. Specifically, we asked whether these two groups exhibit different flower landing preferences and flower constancy toward flower colour and size.

## MATERIALS AND METHODS

### Experimental setup

To compare the flower preferences and constancy of flower naive and experienced *Eristalis tenax* (Linnaeus 1758) (Syrphidae), we used reared naive individuals and experienced hoverflies collected in their native environment, to which we presented artificial flowers offering a steady supply of sugar solution and differing in flower colour (yellow versus white) and size (large versus small). Both naive and experienced hoverflies were then offered the same combinations of treatments, resulting in a fully factorial experimental design for each hoverfly group, which allowed us to compare the results.

### Artificial flowers

**Fig. 1.**
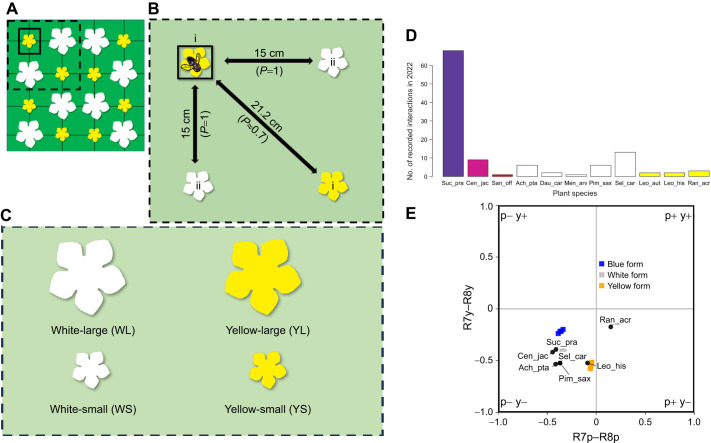
**Experimental design overview.** (A,B) Illustration of the experimental arena and arrangement of the artificial flowers (note, the illustration is not to scale; for a true-to-scale illustration, please see Fig. S6). i and ii placed in the flowers refer to distinct flower forms used in the experiment. (C) Illustration of the tested flower forms: white–large (WL), white–small (WS), yellow–large (YL) and yellow–small (YS). (D) The number of recorded interactions of *Eristalis tenax* with flowering plants at the locality where the experienced hoverflies were collected. The colour of the bars roughly corresponds to the colour of the flowers as seen by the human eye. Suc_pra, *Succisa pratensis*; Cen_jac, *Centaurea jacea*; San_off, *Sanguisorba officinalis*; Ach_pta, *Achillea ptarmica*; Dau_car, *Daucus carota*; Men_arv, *Mentha arvensis*; Pim_sax, *Pimpinella saxifrag*; Sel_car, *Selinum carvifolia*; Leo_aut, *Leontodon autumnalis*; Leo_his, *Leontodon hispidus*; Ran_acr, *Ranunculus acris*. (E) Colour loci in the colour space of white and yellow forms of the artificial flowers and selected flowering species in the locality in the visual model for *E. tenax*, based on Kelber (2001). R7y and R8y represent the photoreceptor cells sensitive to UV/blue and green wavelengths, respectively. p+/− and y+/− represent the neural colour-opponent outputs, calculated as the difference in excitation between the top (R7) and bottom (R8) photoreceptors within the ‘pale’ and ‘yellow’ ommatidial pathways.

We designed 3D models of artificial flowers using the online tool OnShape (https://www.onshape.com). Flower models had five petals and a central disc, which carried a vial with 3% sucrose. The artificial flower was designed to maintain a stable upstream flow of the sucrose solution, resulting in lower variation in the level of available reward between flowers, following the protocol from Ishii and Masuda (2014). Flower models were designed in two size categories with a large variant with a 50 mm diameter and a surface area of 10 cm^2^ and a small variant with a diameter of 30 mm and a surface area of approximately 4.5 cm^2^ (see Figs S1 and S2 for a more detailed description of the artificial flowers; the model can be found online at https://sketchfab.com/jakubstenc). The models were then printed from PLA filament of three colours: white, yellow and blue (FLM-PLA-175-WHT, PLA Pineapple Yellow and PLA Azure Blue, respectively, Prusa Research, a.s., Prague, Czech Republic) on a Prusa i3 MK3S+ 3D-printer (Prusa Research, a.s.; the reflectance spectra are presented in Fig. 1). To test the preferences and flower constancy of *E. tenax*, we used four forms combining the difference in colour and size: yellow–large (YL), yellow–small (YS), white–large (WL) and white–small (WS).

### Experimental arena

For experiments with both naive and experienced hoverflies, artificial flowers were placed into the experimental arena in the cage made from thin transparent net material (80×80×80 cm for naive and 140×140×140 cm for experienced hoverflies). A combination of two flower forms (i.e. treatment) was arranged in a pattern of 16 individual flowers (8 of each form) spaced 15 cm apart, thus not allowing hoverflies to crawl from one flower to another without active flying (Fig. 1A,B). This arrangement differs from a standard chessboard pattern (where all nearest neighbours are different) by introducing variable local neighbourhoods. While edge positions mimic a chessboard, requiring longer flights for constancy, central positions offer same-form neighbours at the minimum distance. To control for the resulting differences in flight distance and visual apparency, we calculated the ‘angular area’ of all available flowers from the hoverfly's perspective and included it as a covariate in our statistical models (see ‘Data analysis’, below). All the flowers were mounted on wooden sticks at a height of 25 cm from the ground covered with green to simulate a green background (see the position in dipteran visual space in Fig. 1E). All flowers were presented in the same horizontal plane at the same height and facing up to allow landing and feeding.

Pollinator foraging activity was recorded using a camera (Lamax W9.1, elem6s.r.o., Prague, Czech Republic). The camera was placed above the flowers with a clear view of the flowers and the surrounding arena, allowing us to visually track the foraging bout of an individual hoverfly. The video records were analysed visually, recording the foraging bout of pollinator visits and times. Unfortunately, we were not able to clearly distinguish individual sexes. Therefore, we did not analyse the effect of sex on pollinator behaviour.

### Experiment with naive *Eristalis tenax*

Similar to Matoušková et al. (2023), we acquired overwintering gravid females of *E. tenax* at the Alkazar Quarry, Central Bohemia, Czech Republic (49.9506N, 14.1240E, WGS 1984) during the winter months of 2021. Females of *E. tenax* were acclimated to room temperature in small cages and consequently laid eggs. Following the standard protocol from Nicholas et al. (2018), eggs were transferred to buckets with diluted rabbit dung (diluted 3:1 with water). After development from eggs to larvae and to adults, individuals of *E. tenax* were kept in a rearing cage in the constant environment under a SunLux UV 150 W PAR38 UV-Vis lamp (SunLux, s.r.o., Brno, Czech Republic), with access to water, sugar and pollen. In total, 115 individuals from two clutches were successfully reared. Adult individuals were kept for approximately 2 weeks in cages prior to the experiments in a room with stable temperature (∼24°C) and light conditions, the same conditions we then used for the experiments.

To train the hoverflies to feed from the artificial flowers, the hoverflies were provided with blue flowers 2 days before the experiment, distinct from flowers used in the treatments. Thus, the naive individuals had enough experience to recognise artificial flowers, but were naive with respect to the flowers from the natural community and flowers used in the treatments. After the pre-training phase, 15 randomly chosen individuals were released into the experimental arena with a particular combination of floral forms (treatment). After the experimental run, the individuals were removed from the arena and placed in the rearing cage with a supply of water, sugar and pollen. No individual was used in treatments twice per day, but all individuals were used the following day, again randomly selected for the treatment. During the day, six experimental runs were conducted in three time intervals in two experimental arenas simultaneously (08:30–10:00 h, 10:30–12:00 h and 12:30–14:00 h). Each treatment was repeated three times during four experimental days (29 December 2021–1 January 2022) with one replication per time interval. Exceptionally, treatment WL versus WS was repeated in two additional runs because of low foraging activity in the previous runs.

### Experiment with experienced *E. tenax*

In August of 2022, we collected foraging individuals of *E. tenax* from a species-rich plant–pollinator community in Central Bohemia near Vernýřov village (49.8466N, 15.1498E WGS 1984). The locality consists predominantly of an agriculturally managed (mowed twice per year) grassland community, rich in flowering plant species. A closer locality description can be found in Janovský et al. (2013). Adult individuals were carefully caught with an insect net during foraging and then placed in a small transport cage in the afternoon (16:00–17:00 h, prior to the decrease of pollinator activity at the locality; see Štenc et al., 2023) prior to the experiment. The next morning, collected individuals were released into the two experimental arenas placed in the locality. Their behaviour was recorded on cameras from the morning hours (∼09:00 h) until the end of pollinator activity (∼17:00 h). In the arena, hoverflies had access to additional water supply, and after the experiment, they were released to different localities to avoid pseudo-replication. All the experimental runs were conducted under sunny weather and no or moderate wind for 6 days in total (11 August 2022–16 August 2022).

### Ethical note

No approval to work with individuals of *E. tenax* is required under the legislation of the Czech Republic. We avoided any unnecessary harm to the individuals of *E. tenax* during all stages of the research.

### Data analysis

The visual analysis of pollinator foraging behaviour from video records resulted in the distinction of ‘visits’, ‘flights’ and ‘bouts’ during the foraging of *E. tenax.* A visit was defined as a clear landing on the upper part of the artificial flower. The flight was then the connecting movement between two sequential visits, and the foraging bout consisted of all the visits and connecting flights between visits. In all the visits, we recorded the identity of the visited flower and its position in the arena, resulting in distinction of the flights between the same or different flower forms. The foraging bout was then characterised by the time of start and end length (in terms of both time and the number of visited flowers). The foraging bout ended if the hoverfly exited from the view for a time longer than 5 s. Multiple foraging bouts from one individual were probably recorded. However, we were not able to control for the identity of individuals and thus we treated the bouts as independent.

All analyses were conducted in R language and environment for statistical computing (https://www.r-project.org/). The visual model was rendered with the pavo package (Maia et al., 2019).

#### Pollinator preference

We measured pollinator preference as the proportion of visited flower forms from the foraging bout. First, we tested preferences within each specific treatment using separate binomial tests (H0=*E. tenax* does not prefer either of the two tested flower forms within the treatment), with one tested flower form in the treatment coded as ‘success’ and the other one as ‘failure’. Second, to test the global effect of flower traits, we pooled the data for each group and calculated two separate quasi-binomial generalised linear models (GLM), one for naive and one for experienced hoverflies, to test the effect of colour, size and the number of traits the flower forms differed in, i.e. the difference in colour was coded as 1; the difference in colour and size was coded as 2. Because we did not conduct experimental runs where hoverflies could choose only one flower form in the arena (i.e. YL versus YL, WS versus WS, etc.), but GLM required these data for complete factorial design, we simulated pollinator decisions in the treatments with the same flower forms in the experimental arena, i.e. situations when pollinators cannot distinguish between forms and randomly choose to visit flowers in the arena. For the simulation, we used the average number of visits per foraging bout and 50% probability of ‘success’ for each visit. The simulated data were generated by the RandVec function from the Surrogate package (https://CRAN.R-project.org/package=Surrogate). We performed the same analysis for both naive and experienced hoverflies separately but using the same methodology. Comparisons between the two groups were subsequently based on the distinct patterns of significance observed in these separate models and the inspection of non-overlapping 95% confidence intervals, rather than a single integrated statistical model.

#### Flower constancy

To test the flower constancy of hoverflies toward individual flower forms, we used a GLM with a binomial distribution. Our response variable, flower constancy, was defined as the proportion of consecutive visits where a hoverfly chose the same flower form as its previous visit, indicating constancy.

As predictors, we included flower colour, size and trait differences (similar to those used for preference tests). To account for the spatial arrangement of flowers in the arena, we introduced the angular area of flowers as a control variable. In this context, the angular area refers to the apparent size of a flower as viewed by the hoverfly from its position in the arena. We calculated this by assuming each flower could be represented as a circle, and the hoverfly could see all flowers from a vantage point 5 cm above the currently visited flower while hovering over it (see Fig. S4).

To include this in the model, we computed the ratio between the total angular area of the currently visited flower form and the total angular area of the alternative flower form present in the arena (Figs S5 and S6). This ratio reflects how much of the hoverfly's visual field is occupied by the visited flower form compared with the other form, depending on the hoverfly's location. In the analysis, we used this ratio to model how the visual dominance of one form over another might influence hoverfly decisions.

We then used this model to predict outcomes for two scenarios: (1) the minimal ratio of angular areas possible in the flower form combination, and (2) the maximal ratio of angular areas possible in the flower form combination. The minimal and maximal angular area ratio differs with the combination of the flower sizes, with values for the minimal scenario of 0.1, 0.24 and 0.47 for combinations from small to large, from same-to-same size and from large-to-small flower, respectively. For the maximal scenario, the ratios were 0.19, 0.39 and 0.65 for combinations from small to large, from same-to-same size and from large-to-small flower, respectively (Table S1). The model considers statistically significant three-way interactions among predictors, as the four-way interaction was not statistically significant.

### Declaration of generative AI and AI-assisted technologies in the writing process

During the preparation of this work the authors used Grammarly and Gemini in order to improve the grammatical structure of the manuscript. After using this tool, the authors reviewed and edited the content as needed and take full responsibility for the content of the publication.

## RESULTS

In total, we recorded 5270 and 31,973 visits of *E. tenax* individuals (mean±s.e.m. 15.95±0.82 and 9.25±0.17 visits per individual, respectively) to flowers exhibited during 429 and 4129 foraging bouts for naive and experienced individuals, respectively. Visits and foraging bouts were not distributed evenly among treatments: in general, naive individuals of *E. tenax* visited more flowers per bout than experienced individuals (on average 12 and 8, respectively; Table S1). Similarly, individual flower forms differed in the number of visits by hoverflies, with slightly more visits toward yellow forms by naive hoverflies (YL: 1829, YS: 1682, WL: 693, WS: 637), but quite balanced in the case of experienced hoverflies (YL: 8000, YS: 7855, WL: 8857, WS: 7261).

### Preference

**Fig. 2.**
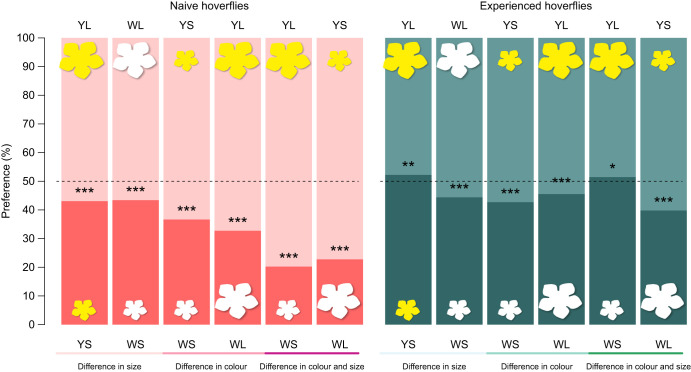
**The preferences of naive (left) and experienced (right) individuals of *E.*** ***tenax*****.** Preferences were derived from binomial tests (Table S2). The letters and images refer to the flower forms compared with each other. Statistically significant preferences are marked by asterisks: ***P<0.001, **P<0.01, *P<0.05.

Both naive and experienced hoverflies showed statistically significant preferences (Table 1); they differed in their magnitude and preference for particular floral forms (Fig. 2). Naive individuals strongly preferred yellow flowers (both large and small), especially when comparing the YL form with the WS form. Flower size itself affected preferences only mildly (Fig. 2; Table S2). Although the visual trends in Fig. 2 appear additive, the GLM analysis (Table 1) revealed a statistically significant interaction between colour and size (*P*<0.001), indicating that the strength of the size preference varies depending on the flower colour. In contrast, experienced hoverflies showed overall lesser (but still statistically significant) preferences when compared with naive hoverflies (Fig. 2; Table S2). Interestingly, experienced hoverflies showed a preference toward the WS form compared with the YL form, which is in contrast with the preferences of naive hoverflies.

**Table 1.** The results of generalised linear models with quasibinomial distribution for naive (left) and experienced hoverflies (right), testing the effect of flower colour, size and trait differences on hoverfly preferences

	Naive hoverflies			Experienced hoverflies		
Predictor	Deviance	d.f.	*P*-value	Deviance	d.f.	*P*-value
Colour	**133.39**	**548**	**<0.001**	**71.90**	**5621**	**<0.001**
Size	**10.36**	**547**	**<0.001**	**15.16**	**5620**	**<0.001**
Diff.	10.76	544	0.09	1.66	5617	0.734
Colour:Size	**14.22**	**543**	**<0.001**	**22.70**	**5616**	**<0.001**
Colour:Diff.	**434.80**	**540**	**<0.001**	**98.77**	**5613**	**<0.001**
Size:Diff.	**18.97**	**537**	**<0.001**	**150.90**	**5610**	**<0.001**
Colour:Size:Diff.	**17.20**	**534**	**<0.001**	**49.67**	**5607**	**<0.001**

The significant predictors are in bold. Predictor ‘diff.’ stands for difference in traits. Link function: logit, residual deviance for naive hoverflies=1003.6 and 534 residuals d.f.; residual deviance for experienced hoverflies=8700.3 and 5607 residual d.f.

### Constancy

**Fig. 3.**
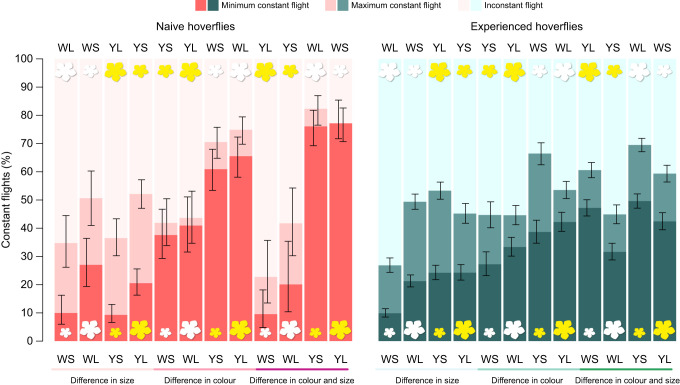
**Constancy of naive (left) and experienced (right) hoverflies based on their flights between visited flower forms.** The letters and images refer to the flower forms compared with each other, with the starting form in the lower row and the compared form in the upper row. The lower and upper part of the bar plot refer to the constancy in the scenario with the lowest and highest ratio of angular areas of the flower forms (the same versus the other) in the arena, respectively. Hence, the difference between minimal and maximal values can be seen as a rough estimate of the effect of the flower's spatial distribution on *E. tenax* constancy. The whiskers correspond to the 95% confidence interval derived from the model.

Naive individuals of *E. tenax* exhibited statistically significant positive flower constancy (staying on the same form more frequently than chance) only when the floral forms differed in colour (Fig. 3, Table 2). When the flower forms differed only in size, pollinators did not show significant positive constancy with any offered flower form. Instead, their behaviour was driven by innate preference and visual apparency (angular area), leading to frequent switching, particularly from small to large flowers, rather than constancy toward a single form. Consequently, differences in flower colour and size had only a limited additive effect on flower constancy, suggesting that flower colour is the main driver of flower constancy of naive *E. tenax* (Table 2). This is supported by the GLM analysis (Table 2), which revealed that although size and interaction effects were statistically significant, their contribution to model deviance was substantially lower (deviance: 16.82 and 5.49, respectively) compared with that of flower colour (deviance: 169.39), identifying colour as the primary driver of constancy. Generally, hoverflies exhibited stronger flower constancy toward yellow flower forms. Flower constancy of experienced hoverflies, similar to that of naive hoverflies, was highest when flower forms differed in colour. In contrast to naive hoverflies, however, flower size statistically significantly affected the flower constancy of experienced individuals. Overall, the flower constancy of experienced hoverflies was about 10–15% lower than that of naive individuals (Fig. 3).

**Table 2.** The results of generalised linear models with binomial distribution for naive (left) and experienced hoverflies (right), testing the effect of flower colour, size, difference in traits and ratio of angular distances of individual flower forms on hoverfly constancy, based on flights between the flower forms regarding the previous visit

	Naive hoverflies			Experienced hoverflies		
Predictor	Deviance	d.f.	*P*-value	Deviance	d.f.	*P*-value
Colour	**169.39**	**1**	**<0.001**	**394.04**	**1**	**<0.001**
Size	**16.82**	**1**	**<0.001**	**21.42**	**1**	**<0.001**
Diff.	**563.65**	**2**	**<0.001**	**845.57**	**2**	**<0.001**
Ang. dist. ratio	**42.90**	**1**	**<0.001**	**201.29**	**1**	**<0.001**
Colour:Size	**5.49**	**1**	**<0.001**	**39.51**	**1**	**<0.001**
Colour:Diff.	**172.61**	**2**	**<0.001**	**8.00**	**2**	**<0.001**
Colour:Ang. dist. ratio	0.41	1	0.52	2.64	1	0.104
Size:Diff.	**51.90**	**2**	**<0.001**	**690.28**	**2**	**<0.001**
Size:Ang. dist. ratio	0.15	1	0.697	**67.09**	1	**<0.001**
Diff.:Ang. dist. ratio	**41.35**	**2**	**<0.001**	**68.63**	**2**	**<0.001**
Colour:Size:Diff.	**6.78**	**2**	**<0.001**	**137.05**	**2**	**<0.001**
Colour:Size:Ang. dist. ratio	0.30	1	0.585	**7.44**	1	**<0.001**
Colour:Diff.:Ang. dist. ratio	6.04	2	0.05	4.73	2	0.09
Size:Diff.:Ang. dist. ratio	**12.09**	**2**	**<0.001**	**10.53**	**2**	**<0.001**

The significant predictors are in bold. Predictors ‘diff.’ and ‘ang. dist. ratio’ stand for difference in traits and angular distance ratio, respectively. Link function: logit, residual deviance for naive hoverflies=5621.1 and 4819 d.f.; residual deviance for experienced hoverflies=35,433 and 27,823 d.f.

The angular area ratio, i.e. the ratio of visible flower forms from the hoverfly position, significantly affected both naive and experienced individuals of *E. tenax.* Hence, hoverflies were affected by their position in the arena, lowering the constancy in situations where in their visual field there were more visible flowers of the other form. In Fig. 3, we present two extreme scenarios derived from the GLM (Table 2): predictions based on the minimal and maximal ratio of angular areas of visited and non-visited flower forms. The difference between the extreme cases then gives the approximate effect of the spatial distribution of the flower forms. In the minimal scenario, hoverflies tended to lower their constancy, and they were more likely to visit the other flower form than in the situation where they were closer to the same form. The difference between the minimal and maximal scenarios was higher for the experienced hoverflies, suggesting a greater effect of angular area ratio on hoverfly flower constancy.

## DISCUSSION

Our results revealed the expected landing preference of *E. tenax* toward yellow-coloured artificial flowers, which was increased by the large flower size. However, we found that experienced individuals of *E. tenax* showed different levels of landing preference (Fig. 2) and lower flower constancy (Fig. 3) than naive individuals. We further discuss possible mechanisms leading to changes in pollinator landing preferences and flower constancy during foraging as well as possible limitations of our study.

### Flower preference

Naive individuals of *E. tenax* exhibit a strong positive landing preference for yellow flowers, probably due to an innate association with pollen (Lunau and Wacht, 1994). The strong landing preference toward yellow colour is known from previous studies (An et al., 2018; Matoušková et al., 2023) and also from experiments with the closely related species *Eristalis cerealis* (Kandori et al., 2021). In our study, experienced *E. tenax* showed a decreased but not eliminated preference for yellow. It is important to note that because of the large sample size, even minor deviations from random choice (e.g. 48% or 49%) were identified as statistically significant. While these results indicate a non-random distribution of visits, we consider such small effect sizes to be of limited biological relevance compared with the strong preferences observed in naive individuals. This behavioural plasticity mirrors findings in bumblebees, which similarly modify their innate preferences (typically for violet/blue) based on foraging experience (Gumbert, 2000). The broader floral diversity at our study site may also influence this decrease, as *E. tenax* encounters predominantly blue–purple and white, rather than yellow flowers. This underscores the importance of community context in shaping pollinator preferences and potential adaptability to changes (Hill et al., 1997; Whitehead et al., 2019; Ye et al., 2020; Finnell and Koski, 2021).

Recently, complete extinction of innate PER preferences of *E. tenax* has been shown in manipulative experiments combining both visual and chemical stimuli, underlining the importance of a complex mixture of floral traits in the *E. tenax* learning process and preference adaptation to the current resource availability (Rajan et al., 2025). Future studies should explore how shifts in floral community composition affect the landing and PER preferences of *E. tenax* and the limits of hoverfly learning as well as how the combined effect of visual and chemical stimuli shapes foraging behaviour of *E. tenax.*

### Flower constancy

Our findings confirm that *E. tenax* displays lower flower constancy than bees, suggesting a more generalised foraging behaviour. This difference may originate from divergent life histories: unlike eusocial bees, where flower constancy can be driven by social information and colony-level foraging efficiency (Hayes and Grüter, 2023), *E. tenax* is a solitary forager without such constraints. Furthermore, hoverflies balance foraging with other activities such as mate seeking and predator avoidance, which may reduce their focus on floral constancy compared with more specialised pollinators.

Interestingly, our data show that flower constancy in *E. tenax* is primarily driven by colour differences rather than combinations of traits such as colour and size. This differs from findings in bumblebees, where multiple traits increase constancy (Gegear and Laverty, 2005). In *E. tenax*, colour appears to dominate as the key factor influencing constancy. Further experiments should examine the role of additional floral traits, such as nectar guides or flower depth, to determine whether they affect hoverfly constancy in a more complex trait space.

Our results show a lower flower constancy of experienced individuals of *E. tenax* compared with naive flies. The constancy of naive and experienced hoverflies was also affected by the position of the hoverflies within the arena (Table 2). The experienced hoverflies decreased their constancy slightly more when they were closer to the other floral forms than naive hoverflies (Fig. 3), showing a higher degree of opportunism in their choices and more generalised foraging behaviour. However, our results may be partly affected by the low concentration of sucrose in our artificial flowers, which may have led experienced hoverflies to switch forms more frequently in search of better rewards (Chittka et al., 1999), similar to the behaviour of bees when facing low-quality rewards (Grüter et al., 2011). Moreover, artificial flowers used in the experiment did allow easy access for the hoverflies, resulting in minimal handling time necessary to collect the reward and, consequently, a reduced need to specialise. Furthermore, because all artificial flowers offered equal rewards, the lower constancy of experienced flies cannot be attributed to learning within the arena. Instead, it probably reflects a generalised foraging strategy acquired in the wild which persists even when resources are uniform. Thus, future research should explore how reward quality and flower restrictiveness influence constancy in *E. tenax* to complement our results.

### Limitations of the study

Our study provides insight into the foraging behaviour of *E. tenax* and how it can be shaped by experience with the natural flowering plant community. However, our study is limited to only one flowering community, which can be quite dynamic (Janovský et al., 2013). Currently, it is beyond our knowledge to what extent the changes in the community composition may affect foraging behaviour of *E. tenax* and whether the behavioural changes would affect pollen transfer or fitness of *E tenax*. Moreover, our study only tested visual stimuli, but *E. tenax* is known to modify its preferences by combining both visual and chemical stimuli (Rajan et al., 2025). Also, as the primary goal of this study was to compare naive and experienced individuals of *E. tenax* rather than test the effect of particular floral traits, we did not aim to include more than two traits with two levels per trait. We acknowledge that different flower traits and differences in the traits may generate different responses of both naive and experienced individuals.

We also acknowledge methodological differences between the groups, specifically regarding cage sizes and pre-training. While experienced flies were tested in larger field cages (140 cm^3^) compared with the laboratory cages (80 cm^3^) used for naive flies, the spatial arrangement of the flower array (4×4 grid, 15 cm spacing) remained identical. Thus, the immediate decision-making environment regarding flight distances and visual angles was preserved. Additionally, experienced flies were not pre-trained on blue artificial flowers. We omitted this step to avoid overwriting their natural foraging history with recent artificial learning, thereby ensuring we tested the effect of natural community experience rather than laboratory conditioning.

### Conclusions

Our study suggests that individuals of *E. tenax* adjust their foraging strategy based on experience with the flowering community, leading possibly to more opportunistic behaviour in species-rich habitats. This flexibility may have an important effect on the pollen transfer of plants visited by *E. tenax*, possibly allowing them to manipulate hoverfly constancy through floral traits and reward levels. Further investigation into the cognitive constraints of *E. tenax* will enhance our understanding of how experience shapes pollinator foraging behaviour in complex environments.

## Supplementary Material

10.1242/jexbio.251711_sup1 Supplementary information
