# Supplementary material for: Learning to be unfaithful – preferences and flower constancy of Eristalis tenax (Syrphidae) decrease with experience
Source: J Exp Biol. 2026 May 11;229(9):jeb251711. doi: 10.1242/jeb.251711 (PMC13245904; doi:10.1242/jeb.251711)
Supplement: Supplementary information [file jexbio-229-251711-s1.pdf]

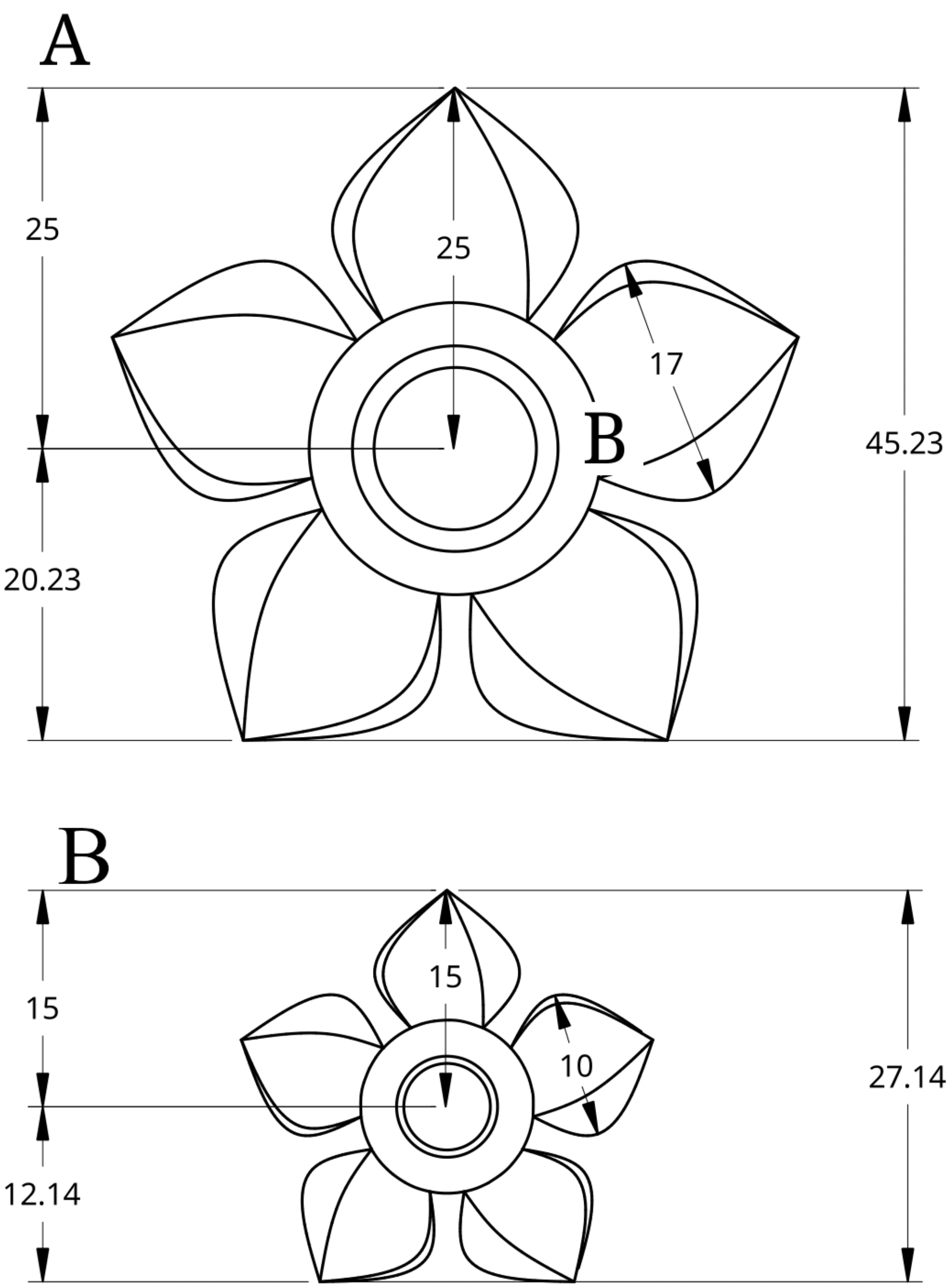

Fig. S1. Technical drawing of different forms of artificial flowers used in the experiment. Numbers indicates dimensions in millimeters. A) Large flower ; B) Small flower

Journal of Experimental Biology • Supplementary information

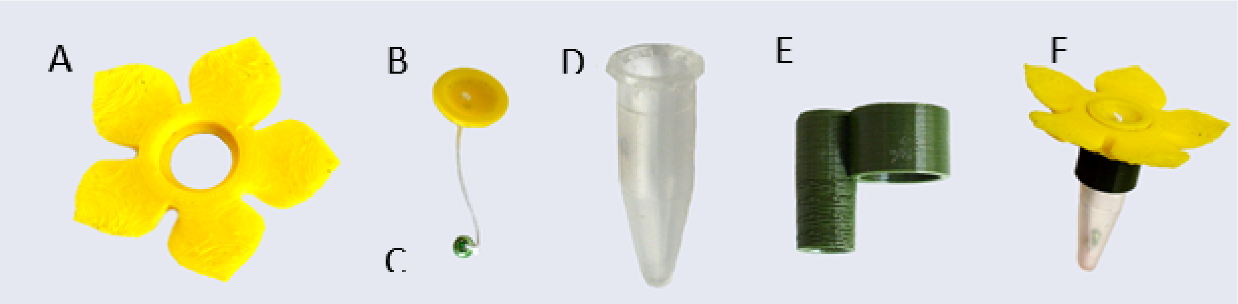

Fig. S2. Part of the artificial flowers. A) artificial corola; B) center of the flower with knot offering the sugar solution; C) wick allowing soaring of the sugar solution; D) Eppendorf tube used as sugar solution container; E) holder of the flower; F) the whole flower. flower.

Journal of Experimental Biology • Supplementary information

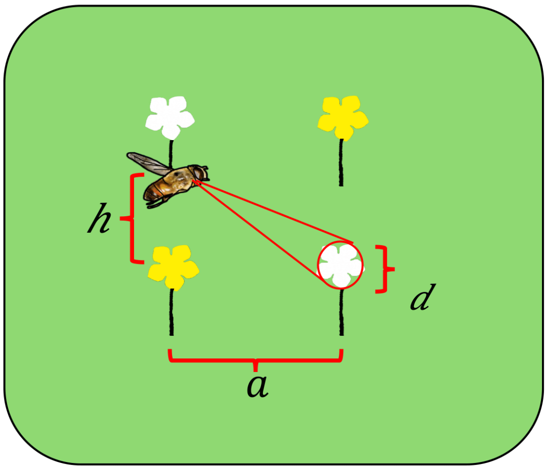

Angular area computation:

$$\alpha = \frac{\frac{\operatorname{atan}(a+\frac{d}{2})}{h} - \frac{\operatorname{atan}(a-\frac{d}{2})}{h}}{2}$$

$$\beta = \operatorname{atan}\left(\frac{\frac{d}{2}}{\sqrt{a^2 + h^2}}\right)$$

$$\text{Angular area} = \pi \times \alpha \times \beta$$

Fig. S3. Illustration of the angular area computation. On the left, we present the variables used in the computation; *h* stands for the height from which the hoverfly observes the array, here assumed to be 5 cm, stands for the Euclidean distance to the flower, and d stands for the diameter of the flower.

Journal of Experimental Biology • Supplementary information

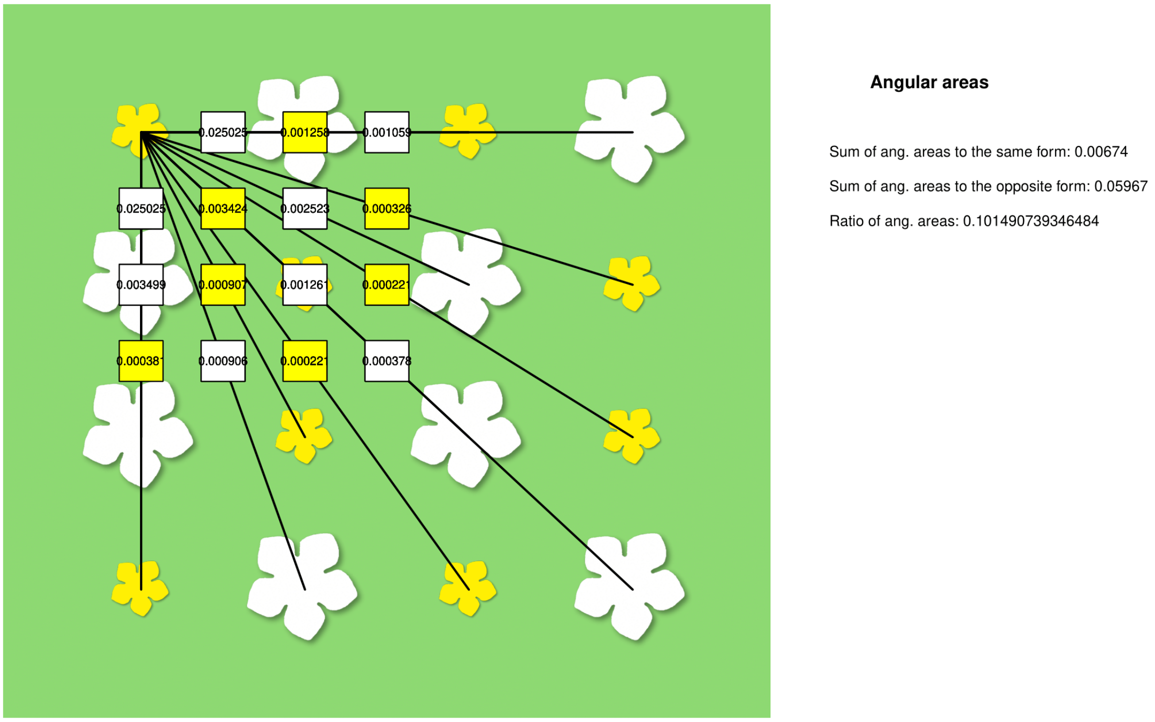

Fig. S4. The arena with flowers of Yellow-Small (YS) and White-Large (WL) forms, demonstrating the angular area of each flower from the 1 position. The values in squares correspond to the angular areas computed according to the equations presented in Fig. 1.

Journal of Experimental Biology • Supplementary information

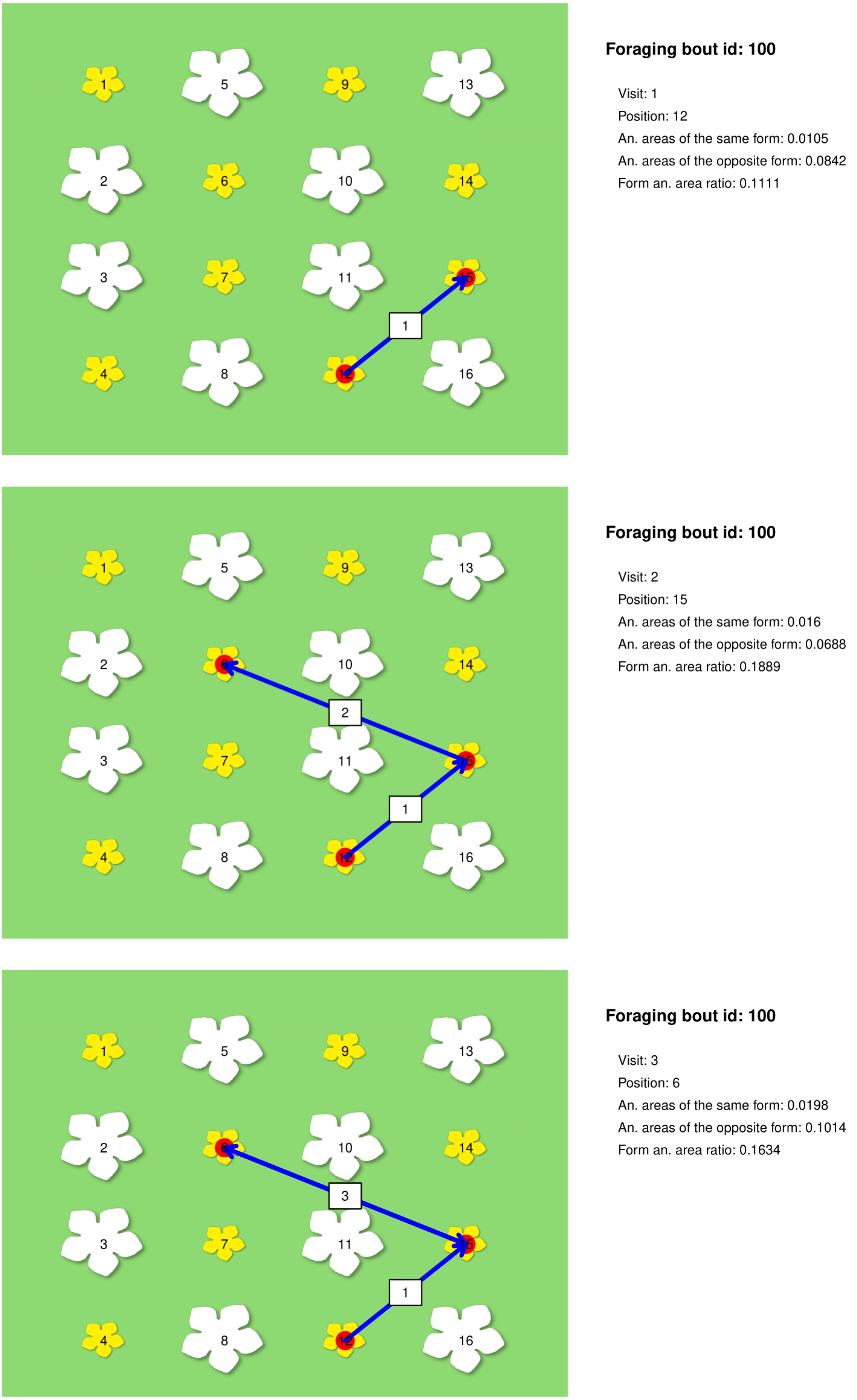

Fig. S5. an example of one foraging bout and the changes of the angular areas with the movement of the fly. A short foraging bout was chosen to provide the simplified image.

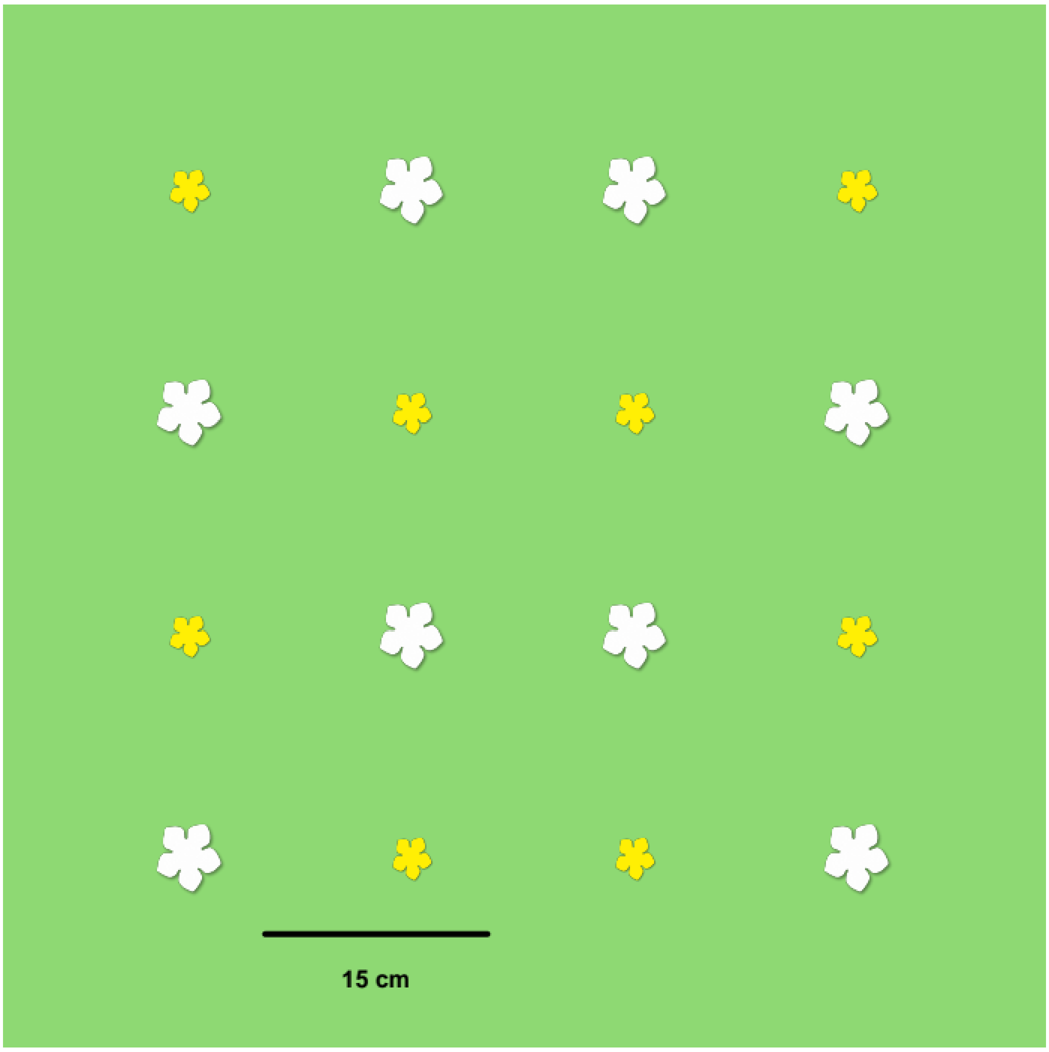

Fig. S6. True-to-scale diagram of the experimental arena. To demonstrate the actual spatial scale experienced by the hoverflies, the flower diameters (Large = 50 mm; Small = 30 mm) and the inter-flower distances (150 mm between centers) are rendered at a strictly proportional scale. The scale bar represents 15 cm.

Table S1. Table of the minimal ratio of angular areas possible in the flower form combination (left side), and the maximal ratio of angular areas possible in the flower form combination (right side). The minimal and maximal angular area ratio differs with the combination of the flower sizes, with values for the minimal scenario 0.1, 0.24 and 0.47 for combinations from small-to-large, from same-to-same size and from large-to-small flower, respectively. For the maximal scenario, the ratios were 0.19, 0.39 and 0.65 for combinations from small-to-large, from same-to-same size and from large-to-small flower, respectively

Min. scenario	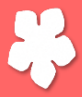	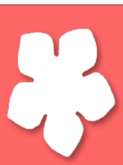	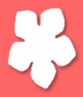	Max. scenario	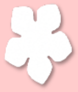	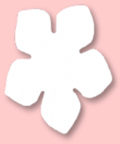	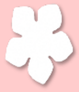
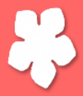	0.24	0.1	0.24		0.39	0.19	0.39
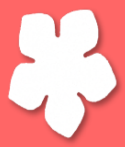	0.47	0.24	0.47		0.65	0.39	0.65

Table S2. Results of the binomial tests for each possible pair of flower forms for naïve (left) and experienced (right) hoverflies. The flower forms are represented graphically, with columns referring to Yellow-Small, White-Large and Yellow-Large, respectively, and rows to White-Small, Yellow-Small and White-Large forms, respectively. Significant preferences are marked as follows: *** p < 0,001; ** p < 0,01; * p < 0,05, green and red colour refers to values higher or lower than 50%, respectively.

	63% ***	57% ***	80% ***		57% ***	56% ***	49% *
		23% ***	57% ***			40% ***	48% **
			67% ***				54% ***

Journal of Experimental Biology • Supplementary information
